# An In Vitro Study on Prestin Analog Gene in the Bullfrog Hearing Organs

**DOI:** 10.1155/2020/3570732

**Published:** 2020-07-02

**Authors:** Zhongying Wang, Minfei Qian, Qixuan Wang, Huihui Liu, Hao Wu, Zhiwu Huang

**Affiliations:** ^1^Department of Otolaryngology-Head and Neck Surgery, Shanghai Ninth People's Hospital, Shanghai Jiao Tong University School of Medicine, Shanghai, China; ^2^Ear Institute, Shanghai Jiao Tong University School of Medicine, Shanghai, China; ^3^Shanghai Key Laboratory of Translational Medicine on Ear and Nose Diseases, Shanghai, China

## Abstract

The prestin-based active process in the mammalian outer hair cells (OHCs) is believed to play a crucial role in auditory signal amplification in the cochlea. Prestin belongs to an anion transporter family (SLC26A). It is densely expressed in the OHC lateral plasma membrane and functions as a voltage-dependent motor protein. Analog genes can be found in the genome of nonmammalian species, but their functions in hearing are poorly understood. In the present study, we used the gerbil prestin sequence as a template and identified an analog gene in the bullfrog genome. We expressed the gene in a stable cell line (HEK293T) and performed patch-clamp recording. We found that these cells exhibited prominent nonlinear capacitance (NLC), a widely accepted assay for prestin functioning as a motor protein. Upon close examination, the key parameters of this NLC are comparable to that conferred by the gerbil prestin, and nontransfected cells failed to display NLC. Lastly, we performed patch-clamp recording in HCs of all three hearing organs in bullfrog. HCs in both the sacculus and the amphibian papilla exhibited a capacitance profile that is similar to NLC while HCs in the basilar papilla showed no sign of NLC. Whether or not this NLC-like capacitance change is involved in auditory signal amplification certainly requires further examination; our results represent the first and necessary step in revealing possible roles of prestin in the active hearing processes found in many nonmammalian species.

## 1. Introduction

Hair cells (HCs) in the cochlea play a critical role in converting mechanical sound waves into neural signals for hearing [1–3]. The mammalian cochlea contains one row of inner hair cells (IHCs) that feed auditory signals to auditory afferent fibers, and three rows of outer hair cells (OHCs) that are able to contract upon depolarization and elongate when hyperpolarized [4–6]. This change of length (electromotility) happens at a microsecond time scale. This form of electromotility surprisingly does not require any force generator like ATP or calcium [4, 7, 8]. It is generally accepted that electromotility provides the physiological basis of a precise frequency selectivity and sensitivity of mammalian hearing [5, 9, 10]. Electromotility is the result of conformational changes of a transmembrane protein named prestin. Prestin belongs to a highly versatile solute carrier 26 (SLC26A) in the anion transporter family [11–13]. Almost all the SLC26A members transport different anion substrates across epithelia, and the mammalian prestin is unique owing to its functions as a voltage-dependent motor protein [13, 14]. The voltage-dependent charge movement conferred by prestin's voltage sensor can be measured as a nonlinear capacitance (NLC) of the cell membrane. The NLC is often used as a substitute for direct measurements of the somatic motility in outer HCs and prestin-transfected cells because it is linked to cell motility and can be easily assayed experimentally [5, 13, 15, 16].

Comparable to that in mammals, the inner ear of nonmammalian vertebrates varies significantly in anatomy across classes. Despite the fact that amphibian hair cells are not as highly differentiated as mammalian OHCs, their ears are also sensitive, sharply tuned, and can spontaneously emit sounds. Both spontaneous and evoked otoacoustic emissions from the American bullfrogs have been reported [17, 18]. The overall emission levels of amphibian ears are larger than those of avian and human ears [19, 20]. The hair bundle and prestin motors in the avian auditory HCs together generate a force underlying amplification and frequency tuning [21, 22]. It remains unclear whether frog HCs have prestin and if frog prestin participates in the active process with the hair bundle.

The American bullfrog has been widely used as an animal model for the study of auditory physiology because of its well-developed middle and inner ear anatomy. The inner ear of the American bullfrog contains three auditory organs: the amphibian papilla (AP), the basilar papilla (BP), and the sacculus (S). The AP receives acoustic stimuli within a frequency range of 100 Hz-1250 Hz, while the BP covers the higher portion of the auditory frequency range from about 1.2 kHz to 4 kHz [23]. The sacculus is a mixed-function organ which is most sensitive to low-frequency sounds (120 Hz ± 24 Hz) and seismic sensation [24, 25]; however, none of these investigations have focused on prestin and electromotility. We generated stable cell lines transfected with the frog prestin by an AAVS1 site-specific integration. The NLC of the frog prestin, both in transfected cells and in primary HCs isolated from frog auditory organs, were measured using a patch-clamp technology. The goal of our work was to investigate whether frog HCs had prestin and if it functioned as an intrinsic motor for amplification and frequency selectivity with the hair bundle.

## 2. Methods

### 2.1. Cloning and Analyses of Prestin Orthologs

We obtained the prestin coding region of gerbil (*Meriones unguiculatus*), tropical clawed frog (*Xenopus tropicalis*), and the American bullfrog (*Rana catesbeiana*) using a BLAST analysis of the Ensembl and NCBI genomic databases. Genomic sequence data from gerbil and bullfrog were used to deduce the full coding cDNAs, which were then synthesized (HuaGene, China). The correct orientation and reading frame were verified by sequence analysis, and ortholog and paralog comparisons were conducted using UniProt, CLUSTALW, and Espript 3. All constructs were verified by gene sequencing.

### 2.2. Generation of Stable Cell Lines That Express fPres and gPres

#### 2.2.1. Construction of Vectors for AAVS1 Site-Specific Integration

The AAVS1 safe harbor locus site-specific integration used CRISPR/Cas9-mediated gene editing. The sgRNA (GGGCCACTAGGGACAGGAT) targeting the AAVS1 site was cloned into a lentiviral vector (pLenti-CRISPR), which contained a SpCas9 expression cassette. A donor vector was generated by assembling PCR-amplified fragments by restriction digestion and ligation. The resulting vector contained two homology arms from HEK293T genomic DNA that flanked an overexpression cassette with a puromycin selection marker on the plasmid backbone (pTOPO-AAVS1-EF1). This donor vector was designed for the expression of fPres- and gPres-enhanced GFP (EGFP) fusion proteins driven by the CMV promoter.

#### 2.2.2. Cell Culture

HEK293T cells were cultured in the Dulbecco's Modified Eagle's Medium (DMEM) (Invitrogen, Carlsbad, CA, USA) supplemented with 10% fetal bovine serum (FBS) (Invitrogen) at 37°C in 5% CO_2_. Mycoplasma testing was performed regularly using PCR detection. Cells were transfected at 60%–80% confluence using the Lipofectamine 2000 DNA transfection reagent (Thermo Fisher Scientific), typically with 2 *μ*g plasmid(s) and 5 *μ*L of the transfection reagent in a 6-well culture dish.

#### 2.2.3. Expression of fPres and gPres in HEK293T Cells

Cells were cotransfected with a mixture of plasmids for sgRNA/Cas9 and the donor (donor : sgRNA/Cas9 = 1.5 *μ*g : 0.5 *μ*g). Then, 2 *μ*g/mL puromycin was added into the culture medium 24 h after transfection and cell pools expressing prestin and EGFP were identified after puromycin screening for 7 d-10 d.

### 2.3. Confocal Imaging

The cells from the stable cell line at passage six were cultured for 12 h before immunodetection. Cells were rinsed with phosphate-buffered saline (PBS) one time and fixed with 4% paraformaldehyde for 30 min. Then, the cells were washed twice for 15 min each before they were permeabilized with PBT (PBS, 1% Triton X-100) and blocked with 1% bovine serum albumin (BSA) in PBS for 1 h at room temperature (RT). Confocal imaging was conducted with a laser scanning microscope (Leica Microsystems, Germany) using a 63x oil immersion objective.

### 2.4. Animals

Adult American bullfrogs (*Rana catesbeiana*) were purchased from a local vendor. Two-week-old C57 mice were purchased from the SIPPR-BK Laboratory Animal Ltd. (Shanghai, China). The care and use of animals were conducted in accordance with the Guide for the Care and Use of Laboratory Animals (National Institutes of Health, USA) and approved by the University Committee of Laboratory Animals of Shanghai Jiao Tong University.

Bullfrogs were sedated in an ice bath for 20 min and then double-pithed and decapitated. Amphibian papillae, basilar papillae, and sacculi were dissected and recorded in an extracellular solution containing (in mM) 95 NaCl, 1 KCl, 1 MgCl_2_, 20 TEA-Cl, 0.5 CaCl_2_, 2 CoCl_2_, and 10 HEPES at pH 7.30 (240 mosmol/L). NaOH was used for pH adjustment.

Cochleae and the apical coil of the organ of Corti were acutely dissected from C57 mice and fixed to a recording chamber. The external solution contained (mM) 120 NaCl, 20 TEA-Cl, 2 CoCl_2_, 2 MgCl_2_, 10 HEPES, and 5 glucose at pH 7.3. NaOH was used for pH adjustment.

### 2.5. Electrophysiology

Recordings of bullfrog HCs were performed at 20°C within 3 h of dissection. Patch pipettes were pulled from thick-walled borosilicate glass (World Precision Instruments) using a Narishige puller (model PP-830) to resistances of 5 M*Ω*–8 M*Ω* and coated with dental wax. Internal solutions for the bullfrog HCs were composed of (in mM) 100 CsCl, 10 EGTA, 10 HEPES, and 1 MgCl_2_ at pH 7.30 (240 mosmol/L). CsOH was used for pH adjustment. Whole-cell voltage-clamp recordings were performed with an EPC-10/2 (HEKA Electronics) patch-clamp amplifier and Pulse software (HEKA). The HCs were held at -80 mV. Offline analysis was performed mainly with the Igor Pro 5.0 software (WaveMetrics).

We recorded mouse OHCs at 20°C within 1.5 h of dissection. Patch pipettes were pulled from thick-walled borosilicate glass (World Precision Instruments) using a Narishige puller (model PP-830) to resistances of about 6 M*Ω* and then coated with dental wax. The internal solution consisted of (mM) 140 CsCl, 2 MgCl_2_, 10 EGTA, and 10 HEPES at pH 7.3. CsOH was used for pH adjustment. The osmolarity was adjusted to 300 mosmol/L.

HEK cells were detached with trypsin (Invitrogen) treatment before recordings were collected. The detached cells were then bathed in an extracellular solution containing (in mM) 120 NaCl, 20 TEA-Cl, 2 CoCl_2_, 2 MgCl_2_, 10 HEPES, and 5 glucose at pH 7.2. Osmolarity was adjusted to 300 mosmol/L with glucose. Recording pipettes were pulled with resistances of 2.5 M*Ω*–5.0 M*Ω* and filled with internal solution (in mM): 140 CsCl, 2 MgCl_2_, 10 EGTA, and 10 HEPES. NLC measurements were performed on cultured cells with a robust membrane-associated EGFP expression. After rupture, we selected the cells whose membrane resistance was over 300 M*Ω* and showed normal *Cm* and *Rm* values.

The sine +DC software lock-in function of Patchmaster was used to obtain the voltage-sensor displacement currents and capacitance; a voltage protocol was designed that included both ramp and sine stimulation (800 Hz with a 10 mV amplitude). Sine waves were superimposed onto ramps from –150 mV to 100 mV for a duration of 300 ms. The NLC was fitted with the derivative of a Boltzmann function:

(1) $$\begin{array}{c} Cm=\frac{{\text{Q}}_{\max}\alpha }{\exp\left[\alpha \left(V_{m}-V_{1/2}\right)\right]{\left(1+\exp\left[-\alpha \left(V_{m}-V_{1/2}\right)\right]\right)}^{2}}+C_{\text{lin}}, \end{array}$$

where *Q*_max_ is the maximum charge transfer, *V*_1/2_ is the voltage at half-maximum charge transfer, *C*_lin_ is the residual linear membrane capacitance, and *α* is the slope factor describing the voltage dependence. *α* = *ze*/*kT*, where *k* is Boltzmann's constant, *T* is the absolute temperature, *z* is the valence of charge movement, and *e* is the electron charge.

## 3. Results

### 3.1. fPres Confers NLC to HEK293T Cells

**Figure 1:**
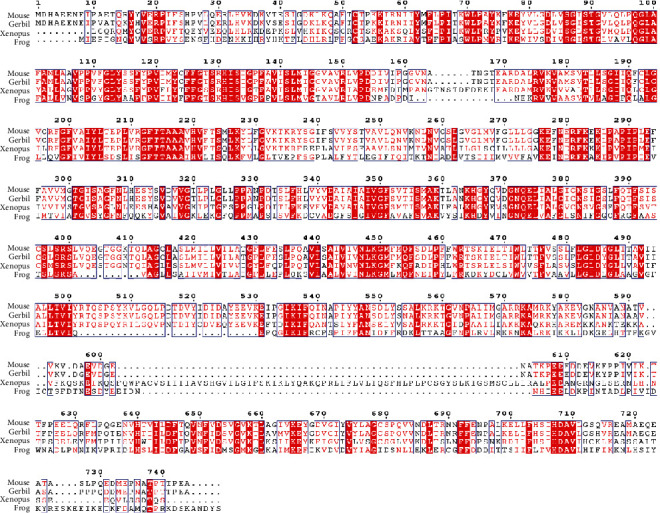
Alignment of amino acid sequences of SLC26A5 of mouse, gerbil, Xenopus, and bullfrog. Different colors had been used to represent identity of each residue among four species. Red block: full identity at a residue; red letter: partial identity at a residue; black: complete disparity at a residue. Gaps in the aligned sequences were indicated by the dashed line.

In order to obtain the prestin coding region of the American bullfrog, we used a BLAST analysis of the Ensembl and NCBI genomic databases. Using the CLUSTAL method, alignment of the mouse, gerbil, *Xenopus*, and *Rana* prestin protein sequences was conducted (Figure 1). This alignment revealed nearly 97% identity among mouse and gerbil, 35% among gerbil and *Rana*, and 57% among gerbil and *Xenopus*. Our alignment results were consistent with former comparative peptide sequence analyses of mammalian prestins that were much more conserved with only minor changes, while prestins were quite variable among vertebrate species like the bony fish, amphibians, and birds [26].

**Figure 2:**
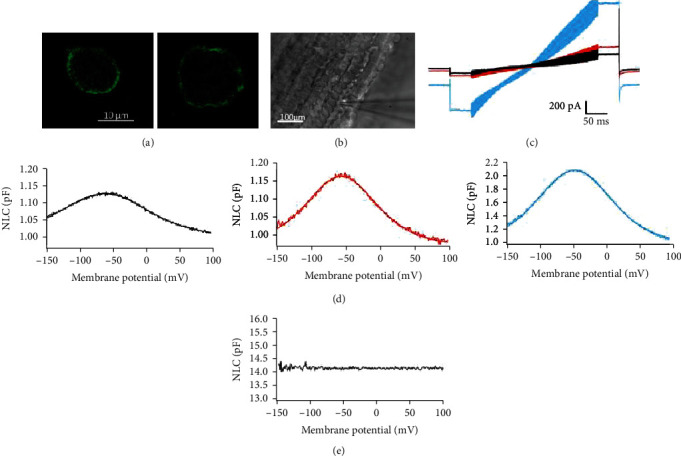
Nonlinear capacitance obtained from gPres- and fPres-transfected cells and a mouse OHC. (a) Confocal microscopy images of HEK cells transfected by gPres and fPres. (b) OHC patch. (c) Whole-cell currents of gPres- and fPres-transfected cells and OHC. Cells were held at -80 mV for current recordings. Voltage steps (300 ms in duration) varied from -150 to 100 mV in 10 mV steps. Black-gPres, red-fPres, blue-OHC. (d) NLC obtained from gPres- and fPres-transfected cells. Black-gPres, red-fPres. NLC obtained from the mouse OHC, blue curve. (e) This one showed the lack of detectable NLC in a representative control cell.

We examined the electrophysiological properties from HEK cells transfected with the fPres-EGFP protein fusions by a site-specific gene transfer at the human AAV site 1 (AAVS1) [27–30]. Transgene expression is influenced by the integration site and some random insertions or transient transfections which can interfere with genes or disturb their transcription, while site-specific integration can minimize variations between different cells and constructs [31, 32]. We chose the gerbil prestin as a positive control, while cells transfected only with the EGFP-vector were a negative control. Membrane expression of fPres and gPres was examined using confocal microscopy. Both the fPres- and gPres-transfected cells showed similar patterns of membrane expression (Figure 2(a)).

Voltage stimulus used for capacitance recordings consisted of a sine wave superimposed onto a voltage ramp. We measured the NLC from the OHCs (Figure 2(b)) and transfected cells.  Figure 2(c) shows the currents of the fPres- and gPres-transfected cells and the OHCs. The fPres-transfected cells had an NLC (the red curve) similar to the bell-shaped curve conferred by the gerbil and mouse prestin (Figure 2(d); black and blue curves). We could not detect NLC in cells transfected only with the EGFP-vector (*n* = 12). An example of a flat response has been presented in Figure 2(e).

Using the first derivative of the Boltzmann function, four parameters (*Q*_max_, *C*_lin_, *V*_1/2_, and *z*) from nonlinear curve fitting of the NLC were calculated. Since the HEK cells varied in size, which is corelated with the *C*_lin_ value, we normalized the *Q*_max_ to the *C*_lin_ to compare the magnitude of the charge movement measured from cells of different sizes. We measured the mouse OHCs as a control.

**Figure 3:**
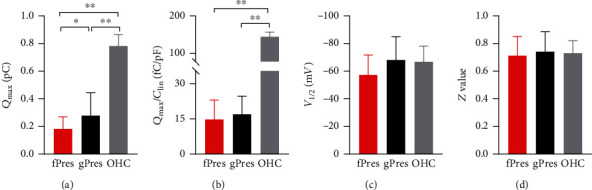
NLC functions of fPres, gPres, and mouse OHC. (a–d) Showed four parameters derived from curve fittings with Boltzmann's function for fPres (*n* = 16), gPres (*n* = 15), and OHC (*n* = 6). Data were expressed as mean ± s.d. ^∗^*P* < 0.05, ^∗∗^*P* < 0.01.

The NLC measurements were analyzed from 15 gPres- and 16 fPres-transfected cells. The means and SEMs of the gPres were *Q*_max_ = 0.27 ± 0.04(fC), *Q*_max_/*C*_lin_ = 16.9 ± 2 (fC/pF), *V*_1/2_ = −68.3 ± 4.4 (mV), and *z* = 0.74 ± 0.04. The means and SEMs of the fPres were *Q*_max_ = 0.18 ± 0.02 (fC), *Q*_max_/*C*_lin_ = 14.9 ± 2.02 (fC/pF), *V*_1/2_ = −58.1 ± 3.5 (mV), and *z* = 0.72 ± 0.03. The means and SEMs of the OHCs were *Q*_max_/*C*_lin_ = 136.4 ± 5.98 (fC/pF), *V*_1/2_ = −71.5 ± 3.6 (mV), and *z* = 0.77 ± 0.03. The magnitude of gPres and fPres NLC was considerably less than that of the OHC (Figures 3(a) and 3(b); *P* < 0.005, Student's *t*-test). The charge density represented by the *Q*_max_/*C*_lin_ was not significantly different between fPres- and gPres-expressing cells; however, the charge density of both transfected cell lines was significantly lower than that measured in OHCs. Another functional parameter of *V*_1/2_ is worth noting (Figure 3(c)). We observed no significant differences in *V*_1/2_ between the gPres- and fPres-transfected cells, or between transfected cells and OHCs. Moreover, there were also no significant difference in the *z* value between gPres, fPres, and the OHC (Figure 3(d)). All the data are shown in Table 1.

### 3.2. NLC Measurements of Frog HCs

**Figure 4:**
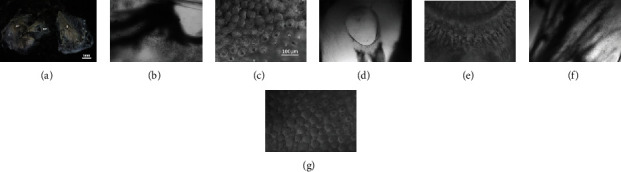
Images of frog's hearing organ. (a) Dissection of the frog's inner ear which contained three auditory organs (AP, BP, and S) under a 10x microscope. (b, c) Displayed was a higher magnification image of the AP under a 100x and 600x microscope. (d, e) Displayed was a higher magnification image of the BP under a 100x and 600x microscope. (f, g) Displayed was a higher magnification image of the S under a 100x and 600x microscope.

The frog inner ear contains three auditory organs: the amphibian papilla (AP), the basilar papilla (BP), and the sacculus (S). The AP is composed of a patch of epithelium covered by HCs. The basilar papilla has a recess opening to the saccular space of the ear. The sacculus is a mixed-function organ which is sensitive to both hearing and vibration. The images of these three auditory organs are shown in Figures 4(a)–4(g). Mammalian, avian, and lizard HCs are located on a basilar membrane. However, the frog inner ear lacks such a sensitive substrate for its sensory cells. Without the basilar membrane, the frog inner ear relies on the tectorial membrane and HCs for frequency selectivity [33].

**Figure 5:**
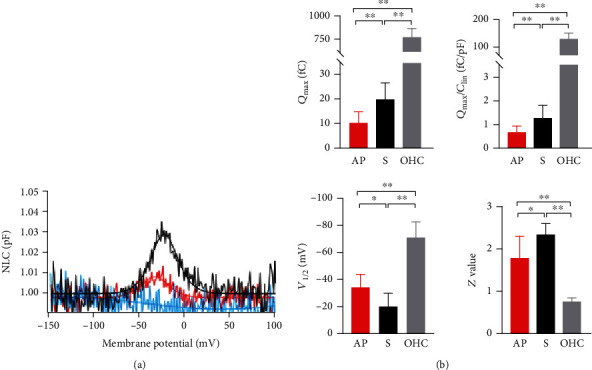
NLC functions of frog's three auditory organs (AP, BP, and S). (a) NLC obtained from the hair cells of three auditory organs. Red: the amphibian papilla (AP); black: the sacculus (S); blue: the basilar papilla (BP). No NLC was detected in the hair cells of the basilar papilla (BP). (b) Four parameters derived from curve fittings with Boltzmann's function for AP (*n* = 10) and S (*n* = 8). Mouse OHC was used as a comtrol. Data are expressed as mean ± s.d. ^∗∗^*P* < 0.01, ^∗^*P* < 0.05 (Student's *t*-test).

We used the same voltage stimulus protocol to record the NLC of the HCs from the AP, BP, and S organs. All AP and S HCs displayed a bell-shaped voltage-dependent NLC (Figure 5(a)). Measurements were analyzed from 10 AP HCs and 8 S HCs (Figure 5(b)). The means and SEMs of the AP HCs were *Q*_max_ = 10.4 ± 1.4 (fC), *Q*_max_/*C*_lin_ = 14.9 ± 1.01 (fC/pF), *V*_1/2_ = −33.8 ± 3.3 (mV), and *z* = 1.8 ± 0.16. The means and SEMs of the S HCs were *Q*_max_ = 19.9 ± 2.4 (fC), *Q*_max_/*C*_lin_ = 16.4 ± 0.68 (fC/pF), *V*_1/2_ = −20.7 ± 3.3 (mV), and *z* = 2.4 ± 0.08. The S HCs had a significant gain of NLC when compared to those from AP (*P* < 0.01). The NLC magnitude of AP and S HCs was significantly less than that of the OHCs (*P* < 0.005, Student's *t*-test), and the charge density of S HCs was significantly higher than that of the AP HCs (*P* < 0.005, Student's *t*-test).

Compared with the mouse OHCs, the charge density of both the AP and S cells was significantly low. The *V*_1/2_ of the AP HCs were more depolarized than that of the S cells (*P* < 0.05), with a difference of approximately 10 mV. The *V*_1/2_ of the OHCs shifted in an even more depolarized direction than that measured in frog cells (*P* < 0.005), with a difference in the *V*_1/2_ between frog HCs and OHCs of about 45 mV. The *z* value of the S HCs was significantly higher than that measured in AP cells (*P* < 0.01), while the *z* values of both AP and S HCs were significantly higher than that of the OHCs (*P* < 0.005).

Notably, we did not observe bell-shaped curves in the BP HCs. As shown in Figure 5(a), the blue curve represents the BP NLC and no evident peak was observed with the voltage applied to AP and S cells; therefore, no fitting results were obtained from the BP cells.

## 4. Discussion

Compared to mammals, many frog species do not have external ears or ear canals. In the frog family, a middle ear cavity is on the medial side of the tympanic membrane, which is coupled to the otic capsule via the stapes. The middle ear transmits acoustic information from the surrounding air to the inner ear, which contains fluid just like those of other vertebrates. Three distinct auditory organs are enveloped in this fluid-filled space: the amphibian papilla, the basilar papilla, and the sacculus. Low-frequency neurons that sense frequencies below 100 Hz innervate the sacculus, mid-frequency neurons that sense frequencies from 100 to 1000 Hz innervate the amphibian papilla, and high-frequency neurons that sense frequencies over 1000 Hz are connected to the basilar papilla [34].

The mammalian ear has frequency selectivity properties due to the propagation of an active traveling wave on the basilar membrane. In the mammalian inner ear, HCs are vulnerable to several forms of damage, including ototoxic drugs, inflammation, and aging [35–40]. The HCs play a critical role in converting mechanical sound waves into electrical signals along the pathway through the spiral ganglion neurons to the cochlear nucleus [41]. The inner HCs serve as sensory receptors, and the outer HCs have the ability to improve cochlear sensitivity and frequency selectivity [10]. Together, they form the basilar membrane–OHC–tectorial membrane complex. What is unique to frogs is that no basilar membrane is attached to their auditory organs. There are no differentiated populations of HCs as there are in mammals. Although there are dramatic anatomical variations between mammals and amphibians, they continue to have many functional similarities. Like mammals, the frog inner ear has a sharp frequency selectivity and can generate both evoked and spontaneous otoacoustic emissions [17]. Since the mechanism found in mammalian ears does not develop in frog ears, additional mechanisms must contribute to the active process of nonmammalian auditory organs.

In order to investigate whether frog prestin was functional, we expressed fPres in HEK293T cells by site-specific gene transferring at the human AAV site 1. Our data showed that fPres produced robust NLC and responded to changes in the membrane potential just like its mammalian ortholog. We used cells transfected with gPres and EGFP alone as positive and negative controls, respectively, to test the functional activity and found that the charge density, *z* value, and *V*_1/2_ of fPres were very similar to that of gPres.

The otoacoustic emissions (OAEs) revealed much about the physiology of the ear. In mammals, OAEs were considered to be the active process generated by the electromotility of the outer HC. The nonmammalian vertebrate inner ear also exhibits an active process, and it is very interesting that the overall emission levels of amphibian ears is the largest, followed by the mammals, and then birds, which have the smallest emission level [19]. In addition to the role of the HC bundle in the active process, we cannot rule out the effect of prestin in amphibian HCs. Since no previous studies have measured amphibian HCs in auditory organs, we did not know if they generated NLC. We used the same voltage stimulus protocol to record HCs isolated from the AP, BP, and S from *Rana catesbeiana*. HCs of the AP and S displayed bell-shaped voltage-dependent NLC, while the cells from BP did not. Notably, our results explained the SOAE test reported by van Dijk et al., who measured SOAE in five frog species, including *Rana catesbeiana*. The highest emission frequency they tested was 1735 Hz, which was within the AP frequency range, and no emissions were recorded in the BP range [17]. It is likely that the prestin expression in the BP HCs was too low to be detected. Another explanation is that the inner ear of frog functions well at a very low frequency. The BP did not act like the AP and S, or even lost its active process ability for its relatively higher frequency sensing range. The *Q*_max_ and charge density of S were higher than that measured in the AP cells. These results may be due to the larger cell size in the S organ, or there might be more prestin expressed in the cell membrane of its tissue. Since charge density directly correlates with the level of prestin expression at the membrane, it is reasonable that S has a larger magnitude of NLC than AP [42].

When we compared the results of frog HCs and mouse OHCs, the charge density was dramatically different between these two taxa. The mouse OHC prestin had a greater charge density than the frog prestin, along with a significant shift of *V*_1/2_ from positive to negative potentials. It is suggested that the functional evolution of prestin lies in the acquisition of NLC and the potential for *V*_1/2_ to shift from positive to negative [26, 43]. Our study supports the hypothesis that the amphibian prestin is evolutionarily less advanced than mammalian prestin.

As we know, there is a charged voltage sensor within prestin that moves through the electrical field and gives rise to an electric current. This electric current, similar to a gating current, generates NLC. The *z* value was quite different between the mammalian and amphibian prestin proteins; however, we did not measure the motility or transport function of the frog prestin in this report. In previous studies, there is a reciprocal trend between NLC magnitude and anion transport properties during the functional evolution of prestin [44]. According to our results that the OHC has more prominent NLC than its nonmammalian orthologs, the transport capability of frog prestin might be stronger and its anion transport capability could be the dominant function of frog prestin. Nevertheless, without direct measurement of motility, the contribution of frog prestin to electromotility cannot be completely ruled out.

## 5. Conclusions

We observed acquisition of NLC both in fPres-transfected cells and in HCs isolated from frog auditory organs. Our results represent the first and necessary step in revealing possible roles of prestin in the active hearing processes found in many nonmammalian species. This might lead to the alternative hypothesis that both prestin and HC bundles might function together as the intrinsic kinetics for amplification and frequency selectivity in amphibian inner ears.

## Figures and Tables

**Table 1:** All the measurements performed in the present study are expressed as mean ± sem.

	*C*_lin_ (pF)	*Q*_max_ (fC)	*V*_1/2_ (mV)	*z*	*Q*_max_/*C*_lin_ (fC/pF)
AP (*n* = 10)	14.9 ± 1.01	10.4 ± 1.4	−33.8 ± 3.3	1.8 ± 0.16	0.69 ± 0.07
S (*n* = 8)	16.4 ± 0.68	19.9 ± 2.4	−20.7 ± 3.3	2.4 ± 0.08	1.25 ± 0.19
fPres (*n* = 16)	12.7 ± 0.91	181.5 ± 22.5	−58.1 ± 3.5	0.07 ± 0.03	14.9 ± 2.02
gPres (*n* = 15)	15.9 ± 1.03	279.4 ± 41.7	−68.3 ± 4.4	0.07 ± 0.04	16.9 ± 2
Mouse OHC (*n* = 9)	5.74 ± 0.14	778.6 ± 26.2	−71.5 ± 3.6	0.77 ± 0.03	136.4 ± 5.98

## Data Availability

The data (data for prestin of bullfrog) used to support the findings of this study are included within the supplementary information file(s).

## Supplementary Materials

Supplementary Materials In our supplementary material, you may obtain all the NLC measurements of the mouse OHCs, HEK293T cells transfected with gPres and fPres, AP HCs, and S HCs from the frog auditory organ.
